# Elevated Plasma Thymic Stromal Lymphopoietin After Acute Myocardial Infarction

**DOI:** 10.3389/fcvm.2022.685677

**Published:** 2022-03-07

**Authors:** Yuhao Zhao, Yeping Zhang, Zongsheng Guo, Zheng Ma, Ye Liu, Chunming Han, Xinchun Yang, Lei Zhao

**Affiliations:** ^1^Heart Center and Beijing Key Laboratory of Hypertension, Beijing Chaoyang Hospital, Capital Medical University, Beijing, China; ^2^Department of Cardiology, Beijing Tongren Hospital, Capital Medical University, Beijing, China

**Keywords:** coronary heart disease, atherosclerosis, acute myocardial infarction, thymic stromal lymphopoietin, major adverse cardiac event

## Abstract

**Background:**

Thymic stromal lymphopoietin (TSLP), a distant paralog of the cytokine IL-7, has been shown to be associated with atherosclerosis. However, the effect of plasma TSLP level after acute myocardial infarction (AMI) remains largely unclear. Thus, we aimed to assess the relationship between the concentration of TSLP at admission and the risk of major adverse cardiovascular events (MACE) in AMI patients.

**Methods:**

A total of 175 patients with AMI and 145 unstable angina (UA) controls were recruited in the present study. The clinical characteristics were collected, and MACE was recorded during hospitalization and the follow-up period after discharge.

**Results:**

The median value (25, 75 percentiles) of TSLP concentrations in the AMI group was higher than that in the UA group [11.18 (8.14–15.22) vs. 8.56 (5.26–11.94) pg/ml, *p* < *0.0*01, respectively]. Multivariate linear regression analysis revealed that Troponin-I (standardized β = 0.183, *p* = 0.004) was an independent factor for TSLP. According to the median of TSLP concentrations, all the AMI patients were divided into the high-level group (TSLP level ≥ 11.18 pg/ml, *N* = 91) and the low-level group (TSLP <11.18 pg/ml, *N* = 84). In a receiver operating characteristic curve analysis, the area under the curve for TSLP as a predictor of AMI was 0.674 with a cut-off value of 9.235 pg/ml. After a median follow-up of 14 months, Kaplan-Meier survival analysis showed no significant difference in MACE-free survival between the two groups (*p* = 0.648). Finally, the multivariate logistic regression analyses demonstrated that TSLP was a negative predictor of MACE in AMI patients (OR:0.778,95% CI:0.733–0.876, *p* = 0.032).

**Conclusions:**

Plasma TSLP levels were elevated in patients with AMI than those in UA. The lower TSLP concentration was associated with MACE after AMI.

## Introduction

Coronary artery disease (CAD) has emerged as a common cause of death over the past few decades. Acute myocardial infarction (AMI) is one of the most serious manifestations of CAD, with a high fatality rate and poor prognosis (1). Therefore, early diagnosis and treatment of AMI are critical. Cardiac troponin is the most common biomarker to identify myocardial injury in clinical practice, but it is commonly affected by a variety of conditions (2). Therefore, novel biomarkers for the diagnosis of AMI are currently under intensive investigation.

Thymic stromal lymphopoietin (TSLP) is a distant analog of the cytokine interleukin-7 (IL-7) and a member of the four-helix-bundle cytokine family (3). TSLP is associated with the pathogenesis of type 2 inflammatory diseases, such as asthma, atopic dermatitis, and inflammatory bowel disease (4–6). These diseases are often complicated by thrombotic events (7–9). TSLP signal is transmitted through TSLP receptor (TSLPR), which is composed of a heterodimer of the IL-7 receptor a chain and the TSLPR chain, and widely distributed in various immune cells, such as B cells, T cells, and mast cells as well as heart tissue (10). Recently, TSLP was reported to play a role in atherosclerosis. Yu et al. found that TSLP was nearly undetectable in cardiovascular tissue of apolipoprotein E-deficient (ApoE–/–) mice. They also found that mice treated with TSLP had significantly fewer atherosclerotic plaques compared with controls, suggesting that TSLP attenuates the development of atherosclerosis (11). Liu et al. reported that TSLP expression was up-regulated in the cardiac tissue and serum of post-MI mice (12).

The most common cause of death in patients with AMI is a malignant arrhythmia or heart failure, which may be closely related to inflammatory responses and is one of the causes of long-term mortality after AMI. However, there are very few studies on plasma TSLP in patients with AMI. Hence, this study aimed to investigate the correlation between plasma TSLP level with AMI and to evaluate the correlation between plasma TSLP level and major adverse cardiovascular events (MACE) after AMI.

## Methods

### Study Subjects

A cross-sectional observational trial was conducted in a single center. All subjects were recruited in the Heart Center of Beijing Chaoyang Hospital, Capital Medical University between May 2019 and November 2019. A total of 175 patients were diagnosed with AMI, and 145 unstable angina (UA) patients as controls were included in the present study. Criteria for AMI diagnosis were as follows: clinical symptoms, typical electrocardiogram changes, elevated cardiac biomarkers (cardiac troponin-I and creatine kinase MB), and coronary angiography. The exclusion criteria were: (1) asthma, (2) autoimmune diseases, (3) pregnancy, (4) neoplasm, (5) severe liver or kidney disease, and (6) severe inflammatory or infectious diseases. The clinical characteristics, including demographic data, history, and echocardiography were collected at the time of enrollment. The study protocol was in accordance with the Declaration of Helsinki and approved by the Ethics Committee of Beijing Chao-Yang Hospital of Capital Medical University. All patients provided written informed consent for participation in the trial.

### Laboratory Measurements

All the blood samples were collected on the first morning after admission under a fasting state from the peripheral vein. Plasma was obtained from the blood samples by centrifugation at 3,000 rpm for 15 min and stored at −80°C, without repeated freeze-thaw cycles until analysis. Plasma concentrations of TSLP were measured using ELISA (Abcam, USA). The operation procedures followed the instructions of the ELISA kit. The intra-assay and inter-assay variations were <5%. The laboratory results, including type b natriuretic peptide (BNP), leukocytes, platelets, troponin I, creatine kinase-MB (CK-MB), C-reactive protein (CRP), erythrocyte sedimentation rate (ESR), D-dimer, and low-density lipoprotein cholesterol (LDL-c), were determined at the clinical laboratory center using standard protocols. The Synergy between Percutaneous Coronary Intervention with Taxus and Cardiac Surgery (SYNTAX) score was calculated to assess the severity of coronary lesions according to the data of coronary angiogram. The Global Registry of Acute Coronary Events (GRACE) risk score is a practical risk assessment tool for in-hospital prognosis.

### Statistics Analysis

Categorical variables were presented as frequencies (percentage) and continuous variables as mean ± *SD* or median and interquartile range (25th and 75th percentiles). Categorical variables were performed using the chi-square test or Fisher's exact test. The two-sample *t*-test and the Mann–Whitney *U*-test were performed for comparisons of continuous variables. The Kolmogorov-Smirnov test was performed to check normality.

Pearson's and Spearman's correlation coefficients were performed to compare TSLP level and clinical variables. Multivariate linear regression analysis and step-wise analysis with forward-selection method (α = 0.05) were performed to identify the variables most strongly associated with TSLP levels in all patients. The univariable with *p* < 0.1 indicated the inclusions in the multivariate model. The Variance Inflation Factor (VIF) test was performed to collinearity diagnosis and logarithmic transformation is used for data conversion of non-normal distribution variables. Clinical events of the all patients were systematically followed-up. The median follow-up time of the present study was 14 (13, 14) months. The primary follow-up endpoint was the occurrence of MACE, including rehospitalization due to cardiovascular diseases and all-cause death. Cumulative event rates were calculated based on Kaplan-Meier survival curves and compared by the log-rank test. A receiver operating characteristic (ROC) curve was utilized to drive the cut-off value of the TSLP level for predicting the AMI. The statistical calculations were performed by IBM-SPSS version 24 (IBM, Armonk, NY, USA). All tests were two-tailed and *p* < 0.05 was considered to indicate statistical significance.

## Results

### Baseline Characteristics

The median value (25th and 75th percentiles) of TSLP concentrations was higher in the AMI group than in the UA group [11.18 (8.14–15.22) vs. 8.56 (5.26–11.94) pg/mL, *p* < 0.001, respectively] (Supplementary Table 1). The non-ST-elevation myocardial infarction (NSTEMI) group had slightly higher plasma levels of TSLP than the ST-elevation myocardial infarction (STEMI) group [12.01 (8.56–17.28) pg/mL vs. 10.4 (7.28–13.61) pg/mL], without significant difference (*p* = 0.248). According to the median value of TSLP concentrations, all patients with AMI were divided into the high-level group (TSLP level ≥ 11.18 pg/mL) (*N* = 91) and the low-level group (TSLP <11.18 pg/mL) (*N* = 84), and the clinical parameters between the two groups were compared. There was no statistical difference in the demographic characteristics between the two groups. The higher levels of TSLP were associated with Hemoglobin (*p* = 0.01). To evaluate cardiac function by echocardiography, plasma TSLP concentration had no significant correlation with left ventricular ejection fraction (LVEF) or baseline left atrial diameter (LAD), left ventricular end-diastolic diameter (LVEDD), or left ventricular end-systolic diameter (LVESD) (Table 1). We further divided the AMI patients into the LVEF>50% and LVEF <50% groups according to LVEF. The median plasma TSLP levels of the LVEF <50% group were lower than the LVEF>50% group [12.01 (8.64–18.46) vs. 10.41 (7.28–15.19) pg/mL, respectively], albeit not significant (*p* = 0.072).

**Table 1:** Basic clinical, laboratory and treatment in AMI patients with higher and lower TSLP levels in plasma.

	**Higher level** **(*N* = 91)**	**Lower level** **(*N* = 84)**	***P***
**Demography**			
Age, years	60 ± 13	62 ± 14	0.399
Male, *n*	77 (84.6%)	67 (79.8%)	0.401
BMI, kg/m2	25.21 ± 2.87	25.99 ± 3.33	0.184
Heart rate, bpm	78 ± 14	77 ± 13	0.973
Systolic blood pressure, mmHg	126 ± 19	128 ± 17	0.59
Diastolic blood pressure, mmHg	73 ± 11	75 ± 11	0.28
Previous MI, *n*, %	23 (25.3%)	16 (19.0%)	0.323
Previous PCI, *n*, %	20 (22.0%)	15 (17.9%)	0.496
Current smoker, *n*, %	40 (44.0%)	41 (48.8%)	0.52
Hypertension, *n*, %	49 (53.8%)	52 (61.9%)	0.281
Diabetes mellitus, *n*, %	29 (31.2%)	27 (32.1%)	0.969
Previous arrhythmia, *n*, %	16 (17.6%)	9 (10.7%)	0.195
Previous stroke, *n*, %	8 (8.8%)	9 (10.7%)	0.685
**Laboratory findings**			
WBC, ×109/L	9.74 ± 3.16	9.54 ± 3.31	0.683
Neutrophil, %	79.6 (69.7, 88.9)	78.5 (67.8, 87.9)	0.743
Lymphocyte, %	15.0 (8.8, 22.2)	14.2 (9.3, 24.1)	0.781
Hemoglobin, g/L	141 ± 17	134 ± 17	0.01
Platelets, ×109/L	217 ± 70	216 ± 60	0.883
Serum albumin, g/L	39.64 ± 4.38	40.40 ± 5.74	0.455
Total cholesterol, mmol/L	4.2 (3.7, 5.3)	4.2 (3.7, 4.9)	0.83
HDL, mmol/L	0.93 (0.74, 1.06)	0.96 (0.82,1.04)	0.5
LDL, mmol/L	2.73 (2.00, 3.36)	2.64 (2.24, 3.27)	0.734
Triglycerides, mmol/L	1.42 (1.02, 2.19)	1.48 (1.02, 2.05)	0.802
Troponin-I, ng/mL	15.65 (4.40, 74.94)	17.96 (2.86, 49.85)	0.872
CK-MB, ng/mL	25.00 (5.30, 105.70)	24.25 (3.20, 80.60)	0.485
BNP, pg/mL	172.0 (75.50, 324.50)	123.50 (63.75,418.25)	0.51
ESR, mm/h	8 (3.5, 18)	9 (4, 16.8)	0.637
C-reactive protein, mg/L	4.71 (2.42, 19.63)	5.66 (2.18, 19.76)	0.978
Serum creatinine, μmol/L	68.9 (61.6, 86.6)	69.1 (60.3, 81.3)	0.484
BUN, mmol/L	5.75 (4.49, 6.84)	5.25 (4.47, 6.61)	0.459
K+, mmol/L	3.9 (3.7, 4.2)	4.0 (3.8, 4.2)	0.446
sTSH, uIU/ml	0.98 (0.45, 2.06)	0.81 (0.53, 1.46)	0.495
D-dimier, mg/L	0.23 (0.19, 0.55)	0.27 (0.19, 0.59)	0.337
Fibrinogen, mg/dL	295.8 (245.2, 353.4)	283.3 (245.2, 364.3)	0.689
SYNTAX score	23.4 ± 10.2	22.5 ± 9.9	0.511
GRACE score	153.0 (134.0, 173.0)	154.0 (130.0, 178.3)	0.656
Echocardiography			
Left atrial diameter, mm	37 ± 5	36 ± 5	0.644
LVEDD, mm	49 ± 5	48 ± 5	0.179
LVESD, mm	32 ± 6	33 ± 7	0.257
LVEF, %	60.0 (48.0 66.0)	60.0 (52, 66)	0.622
**Infarct characteristics**, ***n*****, %**			
STEMI	43 (47.3%)	49 (58.3%)	0.082
NSTEMI	48 (52.7%)	35 (41.7%)	
**Treatment**, ***n*****, %**			
Drug therapy	5 (5.5%)	12 (14.3%)	0.021
PCI	86 (94.5%)	69 (85.2%)	
CABG	0 (0%)	3 (3.5%)	

*TSLP, Thymic stromal lymphopoietin; BMI, body mass index; WBC, White blood count; HDL, high-density lipoprotein; LDL, low-density lipoprotein; CK-MB, creatine kinase MB; BNP, brain natriuretic peptide; ESR, erythrocyte sedimentation rate; BUN, blood urea nitrogen; sTSH, thyroid stimulating hormone; LVEDD, left ventricular end diastolic diameter; LVESD, left ventricular end systolic diameter; LVEF, left ventricular ejection; NSTEMI, non-ST-elevation myocardial infarction; STEMI, ST-elevation myocardial infarction; PCI, percutaneous coronary intervention; CABG, coronary artery bypass grafting*.

### Linear Regression Models for TSLP

We also analyzed the correlation between TSLP level and study variables in all subjects. Plasma TSLP concentrations were associated with CRP (*r* = 0.13, *p* = 0.03), white blood cell count (*r* = 0.203, *p* < 0.001), total cholesterol (*r* = 0.116, *p* = 0.042), LDL-C (*r* = 0.126, *p* = 0.028), Troponin-I (*r* = 0.255, *p* < 0.001), CK-MB (*r* = 0.179, *p* = 0.002), D-dimer (*r* = 0.148, *p* = 0.01) and BNP (*r* = 0.191, *p* = 0.001). Additionally, plasma TSLP level was also correlated with GRACE score (*r* = 0.174, *p* = 0.002) and LVESD (*r* = 0.203, *p* = 0.003). TSLP level was negatively correlated with lymphocytes (*r* = −0.193, *p* = 0.001) and LVEF (*r* = −0.17, *p* = 0.012) (Table 2).

**Table 2:** Association between Plasma TSLP and clinical parameters in UA and AMI patients.

	**r**	***P***
Gender	−0.105	0.06
Age	−0.027	0.631
BMI	−0.05	0.436
ESR	0.023	0.705
C-reactive protein	0.13	0.03
WBC	0.203	<0.001
Neutrophil%	0.177	0.001
Lymphocyte%	−0.193	0.001
Hemoglobin	0.072	0.199
Platelets	0.027	0.634
Fast glucose	0.146	0.011
HbA1C	−0.036	0.544
Total cholesterol	0.116	0.042
HDL-C	−0.077	0.181
LDL-C	0.126	0.028
Triglycerides	0.034	0.554
Homocysteine	0.069	0.42
Uric acid	0.101	0.073
Heart rate	0.024	0.668
Systolic blood pressure	−0.037	0.512
Diastolic blood pressure	−0.085	0.13
BUN	0.043	0.443
Serum creatinine	0.083	0.142
Serum albumin	−0.104	0.068
Troponin-I	0.255	<0.001
CK-MB	0.179	0.002
Fibrinogen	0.028	0.627
D-dimer	0.148	0.01
BNP	0.191	0.001
SYNTAX score	0.113	0.053
GRACE score	0.174	0.002
LAD	0.101	0.138
LVEDD	0.12	0.078
LVESD	0.203	0.003
LVEF	−0.17	0.012

*TSLP, Thymic stromal lymphopoietin; UA, unstable angina; AMI, acute myocardial infarction; BMI, body mass index; ESR, erythrocyte sedimentation rate; WBC, White blood count; HDL, high-density lipoprotein; LDL, low-density lipoprotein; BUN, blood urea nitrogen; CK-MB, creatine kinase MB; BNP, brain natriuretic peptide; LAD, left anterior descending artery; LVEDD, left ventricular end diastolic diameter; LVEF, left ventricular ejection; LVEF, left ventricular ejection fraction; LVESD, left ventricular end systolic diameter*.

Table 3 presents the univariate and multivariate linear regression analyses for TSLP in all patients. We checked the VIF among variables in multivariate analysis and found that Neutrophil percentage and Lymphocyte percentage were collinearity. We removed them from multivariate analysis. Finally, Troponin-I (standardized β = 0.183, *p* = 0.004) was an independent determinant of TSLP.

**Table 3:** Univariate and multivariate linear regression models for TSLP.

**Variable**	**Univariate analysis**		**Multivariate analysis**		**Step-wise analysis**	
	**Standardized β**	***P* value**	**Standardized β**	***P* value**	**Standardized β**	***P* value**
WBC	0.167	0.003	0.017	0.817		
Neutrophil%	0.121	0.031				
Lymphocyte%	−0.150	0.008				
Total cholesterol	0.154	0.007	−0.029	0.854		
LDL	0.133	0.021	0.024	0.882		
Troponin-I	0.215	<0.001	0.259	0.005	0.183	0.004
CK-MB	−0.220	<0.001	−0.103	0.262		
D-dimer	−0.139	0.016	0.150	0.117		
BNP	0.162	0.004	0.039	0.643		
LVESD	0.126	0.030	0.077	0.272		
GRACE score	0.113	0.043	0.047	0.553		

*The forward selection method was used into multivariate linear regression analysis. Non-normal continuous variables were transformed into a logarithmic form. TSLP, Thymic stromal lymphopoietin; WBC, White blood count; LDL, low-density lipoprotein; CK-MB, creatine kinase MB; BNP, brain natriuretic peptide; LVESD, left ventricular end systolic diameter*.

### Circulating TSLP Levels Predict MACE

**Figure 1:**
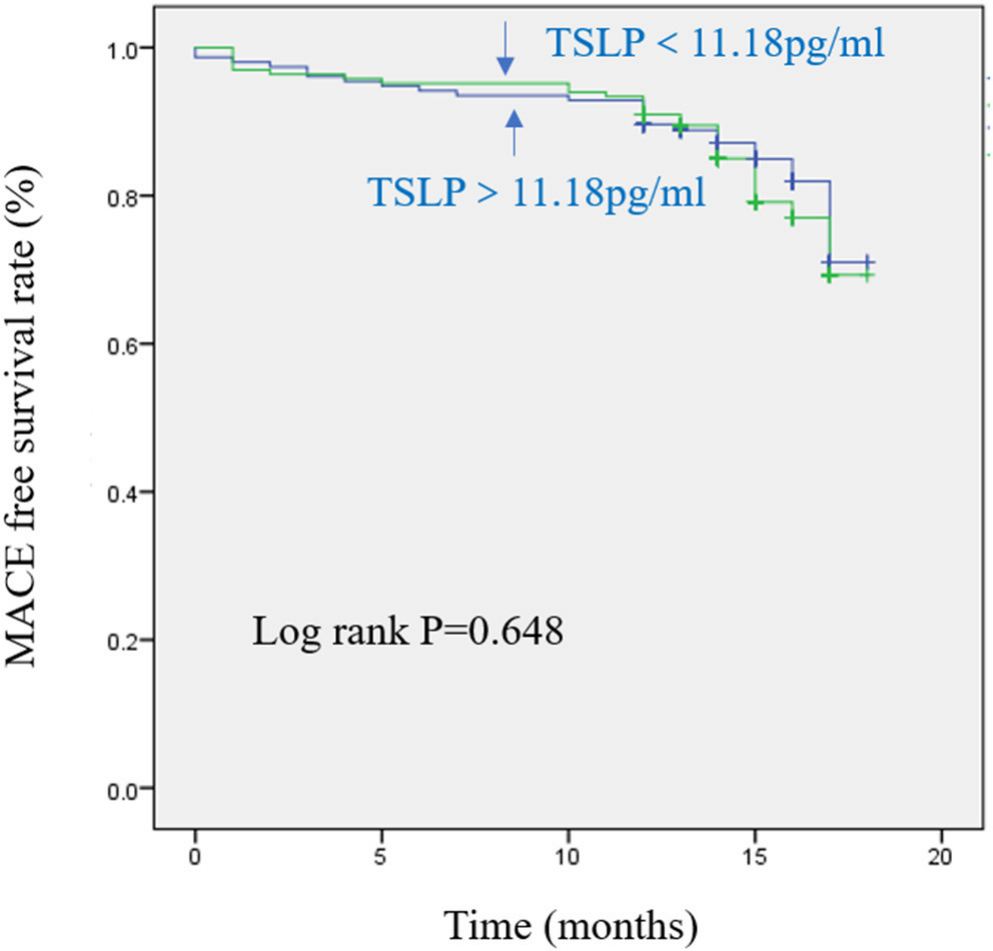
Kaplan-Meier curves in patients with acute myocardial infarction (AMI) with individual levels of thymic stromal lymphopoietin (TSLP) during follow-up.

During a median follow-up of 14 (13, 14) months, 17 patients with AMI (cardiovascular death, *n* = 3) in the high-TSLP group and 13 patients (cardiovascular death, *n* = 2) in the low-TSLP group experienced MACE. The Kaplan-Meier survival analysis showed that there was no significant difference between the two groups (Figure 1).

The ROC curve for TSLP as a predictor of AMI showed an area under the curve of 0.674 (95% CI:0.615–0.733). A cutoff point of 9.235 pg/ml for TSLP had a sensitivity of 65.7% and a specificity of 62.1% in predicting AMI. Among the 175 AMI patients, those with TSLP > 9.235 pg/ml were more likely to smoke (*p* = 0.047) (Supplementary Table 2). Furthermore, we performed binary logistic regression analysis to evaluate the role of gender, age, TSLP, SYNTAX scores, GRACE scores, and LVEF in predicting MACE in AMI patients. The univariate logistic showed that plasma TSLP concentration might predict MACE (OR:0.854, 95% CI:0.745–0.979, *p* = 0.024). This association remained still significant in the multivariate analysis, which showed that TSLP levels were the independent determinants with OR of 0.778 (95% CI:0.733–0.876, *p* = 0.032) (Table 4).

**Table 4:** Univariate and multivariate logistic regression analysis to evaluate TSLP in predicting MACE in AMI patients.

	**Univariate analysis**		**Multivariate analysis**	
	**OR (95%CI)**	***P***	**OR (95%CI)**	***P***
Sex	10.31 (0.63–169.47)	0.102		
Age	0.962 (0.896–1.034)	0.291		
TSLP	0.854 (0.745–0.979)	0.024	0.778 (0.733–0.876)	0.032
SYNTAX score	1.119 (1.035–1.211)	0.005	1.045 (0.967–1.114)	0.007
GRACE score	1.02 (0.988–1.05)	0.245		
LVEF	0.93 (0.877–0.987)	0.016		

*TSLP, Thymic stromal lymphopoietin; MACE, major adverse cardiovascular events AMI, acute myocardial infarction; LVEF, left ventricular ejection; OR, Odds Ratio*.

## Discussion

In the current study, we, for the first time, evaluate the relationship between plasma TSLP levels and AMI, and its clinical outcomes. The data revealed that TSLP plasma levels were significantly higher in AMI patients than in UA patients. Moreover, TSLP plasma levels were associated with inflammatory markers, myocardial injury markers, and BNP in the CAD patients. Based on the previous findings, we speculated that circulating TSLP concentrations might be associated with the MACE in the AMI population.

Thymic stromal lymphopoietin (TSLP), an IL-7-like cytokine, was originally found in the culture supernatant of a mouse thymic stromal cell line that exerts its biological function through the TSLPR (13). This cytokine is a key factor to connect the interface reaction between the body and environment (skin, airway, gut, ocular tissues, etc.) to Th2 responses. TSLP has been implicated to play an important role in allergic diseases, such as asthma, atopic dermatitis, and inflammatory bowel disease (4–6).

Atherosclerosis is a chronic inflammatory disorder characterized by complex immunological interactions between residential vascular cells and professional immunocytes, including T-cells, macrophages, and smooth muscle cells (15). Mortality related to atherosclerosis is mainly caused by cardiovascular events resulting from atherothrombosis. Some studies have proposed the role of TSLP in atherosclerosis, but in part the results are controversial. Wu et al. speculated that TSLP could promote atherosclerotic because the degree of atherosclerosis in TSLPR E-deficient and ApoE–/– mice on a high-fat diet was lower than ApoE–/– controls (16). Another study described that TSLP is strongly expressed in human atherosclerotic plaques (14). In contrast, Yu and co-workers reported that the expression of TSLP in the cardiovascular tissue of ApoE–/– mice was inhibited, and treatment with TSLP could prevent the development of atherosclerosis in these mice (11). Steinmetz et al. showed that TSLP/TSLPR signaling also mediated the atheroprotective effect of Freund's adjuvant in ApoE–/– mice (17). The protective effect of TSLP on atherosclerosis may be related to the down-regulation of systemic inflammation. In this study, the baseline level of TSLP was significantly increased in AMI patients compared with that in UA controls. However, while TSLP is known to be up-regulated after AMI episodes, its role in that state, whether beneficial to contributing to the deterioration of cardiac function or control inflammation, remains unclear. Interestingly, we also found that elevated TSLP level in patients with AMI is protective against MACE. The two important processes in the development of AMI are Plaque rupture and thrombosis. Wang et al. (18) found that TSLPR was expressed on platelets, and its expression was significantly increased in patients with acute coronary syndrome and promoted platelets activation through phosphatidylinositol 3-kinase (PI3K) and its downstream effect Akt. This signal pathway may be involved in plaque rupture and thrombosis in patients with AMI. (19, 20) As an effective protective cytokine in atherosclerosis, transforming growth factor-beta (TGF-β) has attracted extensive attention (21). Yu et al. (22) observed TGF-β was abundantly expressed in plasma, aorta, and spleen of TSLP treated mice. The exact mechanisms of the circulating TSLP association in AMI need further study to elucidate the causality.

Heart failure (HF) is a major factor affecting the prognosis of AMI, with a significant risk of morbidity and mortality. Macrophages are the major responder cells of the immune system after AMI (23). There are mainly two types of macrophages involved in cardiac repair: the typical activated M1 macrophages secrete pro-inflammatory cytokines, including interleukin 6 and tumor necrosis factor-a, and alternatively activated M2 macrophages produce anti-inflammatory cytokines, such as interleukin 4 and interleukin 10 (24, 25). The early phenotypic transfer of M1 to M2 macrophages leads to significant improvements in myocardial infarction injury modeling and cardiac function (26). Liu et al. found that TSLP promoted the polarization of M1 to M2 macrophage, and Ang II-induced TSLP expression in vitro to skew the phenotype of macrophage toward M2 (12). Substantial evidence has demonstrated that the renin-angiotensin system is activated after AMI. Ang II, the central product of the renin-angiotensin system, participates in the development of myocardial remodeling after AMI (27). Liu et al. also reported that Ang II up-regulated the expression of TSLP (12). The activated mast cell-derived proteases and neutrophils after AMI, both of which can convert Ang I to the active Ang II (28), maybe the reason for the elevated TSLP level. Although the circulating TSLP level did not correlate with LVEF after AMI in this study, we have demonstrated that baseline BNP was significantly correlated with plasma TSLP levels. In conclusion, these data suggested that TSLP mediates cardiovascular effects through a variety of mechanisms and can predict the prognosis of AMI.

## Limitations

This study had several limitations. First, plasma TSLP level and MACE are not causally associated. The long-term prognostic value of TSLP for patients with AMI and the underlying mechanisms need to be studied in the future. Second, this was a cross-sectional study conducted in a single-center, with a low evidence-based level. Third, this study had a small sample size; so, a large sample size study is needed to verify our findings. Finally, we collected blood samples at admission and did not have serial plasma TSLP data at follow-up.

## Conclusion

This was the first study to demonstrate that the plasma TSLP concentration was increased in patients with AMI compared with the UA controls. The lower TSLP level is an independent predictor of MACE in AMI patients. Understanding the correlation between TSLP concentration and other established prognostic factors may facilitate the prognosis and management of AMI.

## Data Availability Statement

The raw data supporting the conclusions of this article will be made available by the authors, without undue reservation.

## Ethics Statement

The studies involving human participants were reviewed and approved by the Ethics Committee of Beijing Chao-Yang Hospital of Capital Medical University. The patients/participants provided their written informed consent to participate in this study.

## Author Contributions

XY and LZ: designed the study. YHZ, YPZ, and CH: collected the data and performed the statistical analysis. YHZ, ZG, and ZM: wrote the manuscript. YL, XY, and LZ: revised the manuscript. All authors contributed to the article and approved the submitted version.

## Conflict of Interest

The authors declare that the research was conducted in the absence of any commercial or financial relationships that could be construed as a potential conflict of interest.

## Publisher's Note

All claims expressed in this article are solely those of the authors and do not necessarily represent those of their affiliated organizations, or those of the publisher, the editors and the reviewers. Any product that may be evaluated in this article, or claim that may be made by its manufacturer, is not guaranteed or endorsed by the publisher.
