# Supplementary material for: Elevated Plasma Thymic Stromal Lymphopoietin After Acute Myocardial Infarction
Source: Front Cardiovasc Med. 2022 Mar 7;9:685677. doi: 10.3389/fcvm.2022.685677 (PMC8936131; doi:10.3389/fcvm.2022.685677)
Supplement: Supplementary file 1 [file Table_1.docx]

**Supplementary Table 1.** **The Median value of plasma TSLP level in UA and AMI patients**

|  | **UA (N=145)** | **AMI (N=175)** |  |
| --- | --- | --- | --- |
| **TSLP (pg/ml)** | **8.56（5.26, 11.94）** | **11.18（8.14，15.22）** | **P＜0.001** |

TSLP, Thymic stromal lymphopoietin; AMI, acute myocardial infarction; UA, unstable angina.
