# Supplementary material for: Elevated Plasma Thymic Stromal Lymphopoietin After Acute Myocardial Infarction
Source: Front Cardiovasc Med. 2022 Mar 7;9:685677. doi: 10.3389/fcvm.2022.685677 (PMC8936131; doi:10.3389/fcvm.2022.685677)
Supplement: Supplementary file 2 [file Table_2.docx]

**Supplementary Table 2. Basic clinical, laboratory and treatment in AMI Patients with TSLP levels in plasma according to cut-off point.**

|  | Higher level(N=115) | Lower level(N=60) | P |
| --- | --- | --- | --- |
| Demography |  |  |  |
| Age, years | 61±14 | 61±13 | 0.735 |
| Male, n, | 97(84.3%) | 47(78.3%) | 0.323 |
| BMI, kg/m2 | 25.3±2.8 | 26.2±3.6 | 0.141 |
| Heart rate, bpm | 78±13 | 76±14 | 0.876 |
| Systolic blood pressure, mmHg | 127±19 | 126±16 | 0.694 |
| Diastolic blood pressure, mmHg | 73±11 | 76±11 | 0.127 |
| Previous MI, n, % | 29(25.2%) | 10(16.7%) | 0.197 |
| Previous PCI, n, % | 25(21.7%) | 10(16.7%) | 0.426 |
| Current smoker, n, % | 47(40.9%) | 34(56.7%) | 0.047 |
| Hypertension, n, % | 66(57.4%) | 35(58.3%) | 0.905 |
| Diabetes mellitus, n, % | 38(33.0%) | 18(30%) | 0.682 |
| Previous arrhythmia, n, % | 20(17.4%) | 5(8.3%) | 0.104 |
| Previous stroke, n, % | 12(10.4%) | 5(8.3%) | 0.656 |
| Laboratory findings |  |  |  |
| WBC, ×109/L | 9.7±3.1 | 9.4±3.3 | 0.548 |
| Neutrophil，% | 77.4（69.4,88.8） | 78.8（67.9,88.9） | 0.881 |
| Lymphocyte, % | 13.9（8.8,22.6） | 15.3（9.3,24.1） | 0.756 |
| Hemoglobin, g/L | 138±18 | 135±17 | 0.484 |
| Platelets, ×109/L | 215.3±67.2 | 220.3±61.8 | 0.585 |
| Serum albumin, g/L | 39.6±4.3 | 40.6±6.2 | 0.236 |
| Total cholesterol, mmol/L | 4.3（3.7,5.1） | 4.2（3.8,4.8） | 0.765 |
| HDL, mmol/L | 0.94（0.79,1.05） | 0.92（0.82,1.10） | 0.965 |
| LDL, mmol/L | 2.72（2.08,3.37） | 2.66（2.38,3.24） | 0.725 |
| Triglycerides, mmol/L | 1.44（1.03,2.12） | 1.42（1.00,2.08） | 0.786 |
| Troponin-I, ng/mL | 18.1（3.4,52.7） | 16.5（3.1,89.2） | 0.722 |
| CK-MB, ng/mL | 24.2（4.5,82.5） | 36.1（4.3,96.6） | 0.647 |
| BNP, pg/mL | 169.0（72.0,356.5） | 111.0（60.0,297.0） | 0.264 |
| ESR, mm/h | 8（5,18.5） | 7.5（3.25,15） | 0.377 |
| C-reactive protein, mg/L | 4.71(2.0,20.9) | 5.9(2.5,19.5) | 0.6 |
| BUN, mmol/L | 5.7(4.5,6.9) | 5.3(4.4,6.6) | 0.303 |
| Serum creatinine, μmol/L | 69.0(62.0,86.6) | 69.1(59.9,81.3) | 0.376 |
| K+, mmol/L | 3.97±0.43 | 4.03±0.33 | 0.171 |
| sTSH, uIU/ml | 1.00(0.46,2.07) | 0.80(0.51,1.31) | 0.122 |
| D-dimier, mg/L | 0.26(0.19,0.59) | 0.27(0.19,0.48) | 0.771 |
| Fibrinogen, mg/dL | 296.0(245.2,366.3) | 274.1(245.2,352.3) | 0.294 |
| GRACE score | 156.77±34.31 | 153.15±29.88 | 0.697 |
| SYNTAX score | 23.44±10.15 | 22.06±9.85 | 0.283 |
| Echocardiography |  |  |  |
| Left atrial diameter, mm | 37±5 | 36±4 | 0.235 |
| LVEDD, mm | 48±5 | 47±5 | 0.126 |
| LVESD, mm | 33±6 | 32±7 | 0.161 |
| LVEF, % | 60.0(48.0,66.0) | 60.0(52.0,66.0) | 0.915 |
| Infarct characteristics, n, % |  |  |  |
| STEMI | 57(49.6%) | 33(55%) | 0.495 |
| NSTEMI | 58(50.4%) | 27(45%) |  |
| Treatment, n, % |  |  |  |
| Drug therapy | 12（10.4%） | 6（10%） | 0.478 |
| PCI | 102（88.7%） | 52（86.7%） |  |
| CABG | 1（0.9%） | 2（3.3%） |  |

TSLP, Thymic stromal lymphopoietin; BMI, body mass index; PCI, percutaneous coronary intervention; WBC, White blood count; HDL, high-density lipoprotein; LDL, low-density lipoprotein; CK-MB, creatine kinase MB; BNP, brain natriuretic peptide; ESR, erythrocyte sedimentation rate; BUN, blood urea nitrogen; sTSH, thyroid stimulating hormone; LVEF, left ventricular ejection; NSTEMI, non-ST-elevation myocardial infarction; STEMI, ST-elevation myocardial infarction; CABG, coronary artery bypass grafting.
